# Clinical efficacy of bladder hydrodistension combined with oral mirabegron and intravesical instillation in the treatment of interstitial cystitis

**DOI:** 10.12669/pjms.42.9.16265

**Published:** 2026-09

**Authors:** Lei Du, Ziyue Ma, Wenyong Xue, Wei Li

**Affiliations:** 1 Lei Du, Department of Urology, The Second Hospital of Hebei Medical University, Shijiazhuang 050000, Hebei, China; 2 Ziyue Ma, Department of Urology, The Second Hospital of Hebei Medical University, Shijiazhuang 050000, Hebei, China; 3 Wenyong Xue, Department of Urology, The Second Hospital of Hebei Medical University, Shijiazhuang 050000, Hebei, China; 4 Wei Li, Department of Urology, The Second Hospital of Hebei Medical University, Shijiazhuang 050000, Hebei, China

**Keywords:** Bladder hydrodistension, Intravesical instillation, Interstitial cystitis, Mirabegron, Therapy

## Abstract

**Objective:**

To evaluate the therapeutic efficacy of bladder hydrodistension combined with oral mirabegron and intravesical instillation in patients with interstitial cystitis(IC).

**Methodology:**

This was a retrospective study. Ninety patients diagnosed with IC at The Second Hospital of Hebei Medical University between October 2023 to October 2025 were retrospectively enrolled and randomized into two groups(*n=*45 each). All participants underwent cystoscopic examination followed by bladder hydrodistension. Patients in the control group received oral mirabegron sustained-release tablets in conjunction with hydrodistension. In addition to the same regimen, patients in the study group were administered sodium hyaluronate via intravesical instillation. The treatment duration for both groups was six months. Clinical symptom improvement and clinical efficacy were assessed at the end of treatment.

**Results:**

After six months of treatment, scores on the Pelvic Pain and Urgency/Frequency Patient Symptom Scale, Interstitial Cystitis Symptom Index, and Interstitial Cystitis Problem Index were significantly lower in the study group than in the control group(*p=* 0.00, respectively). The overall response rate was higher in the study group compared with the control group(*p=* 0.03). Patients receiving the triple therapy exhibited a greater reduction in both daytime voiding frequency and nocturia episodes(*p=* 0.00, respectively), together with a significantly increased maximum bladder capacity(*p=* 0.00).

**Conclusion:**

The combination of bladder hydrodistension, oral mirabegron, and intravesical instillation demonstrates superior clinical benefit in patients with IC. This regimen can enhance symptom relief, decrease urinary frequency, expand bladder capacity, and reduce urinary levels of EGF, NGF, and IGF-1 without a significant increase in adverse events.

## INTRODUCTION

Interstitial cystitis (IC), also referred to as interstitial cystitis/bladder pain syndrome (IC/BPS), is a chronic disorder characterized primarily by bladder pain, urinary frequency, and urgency. Epidemiological data indicate a global prevalence of approximately 0.5%–1%, with a markedly higher incidence in women than in men.^1^ In addition to persistent urinary frequency and urgency, patients frequently report suprapubic or perineal discomfort. Pain typically intensifies with bladder filling and is partially relieved after voiding, resulting in substantial impairment in quality of life. The underlying mechanisms include heightened sensory afferent signaling, urothelial barrier dysfunction, and dysregulated cytokine expression, which collectively contribute to disruption of the glycosaminoglycan (GAG) layer and progressive fibrotic remodeling of the bladder wall.^2^

Current management strategies are largely multimodal, involving behavioral interventions, oral pharmacotherapy, bladder hydrodistension, and intravesical instillation of heparin. Although these approaches may yield short-term symptom relief, sustained efficacy is often limited, and relapse is common. Consequently, the development of multi-targeted combination regimens for optimized long-term outcomes remains a clinical priority. Bladder hydrodistension serves both diagnostic and therapeutic purposes and remains a cornerstone intervention. Through mechanical expansion, the bladder can transiently improve bladder compliance; however, when used alone, its therapeutic benefit is frequently modest and short-lived.^3^

This highlights the need for additional interventions. Mirabegron is a β3-adrenergic receptor agonist used to reduce detrusor overactivity. Clinical studies have demonstrated that mirabegron can reduce voiding frequency by up to 40%.^4^ Intravesical instillation of sodium hyaluronate, on the other hand, is intended to restore the integrity of the damaged GAG layer, suppress inflammatory mediator release, and modulate nerve hypersensitivity.^5^ On this basis, this study combined bladder hydrodistension with oral mirabegron and intravesical instillation of sodium hyaluronate, aiming to achieve synergistic therapeutic effects through complementary mechanisms.

## METHODOLOGY

This was a retrospective study. A total of 90 inpatients diagnosed with IC at The Second Hospital of Hebei Medical University between October 2023 to October 2025 were consecutively recruited and randomly allocated to either the study group or the control group(*n =* 45 each). Patient data comes from the electronic medical record system in our hospital. According to the data of each indicator in the pre-survey, the sample size is estimated by 95% confidence interval, and the largest one is the sample size of the study. The sample size required for each group was ≥45 cases on the basis of Fisher exact probability.

### Ethical approval:

The study was approved by the Institutional Ethics Committee of The Second Hospital of Hebei Medical University (No.2025-R827; Date: October 29, 2025), and written informed consent was obtained from all participants.

### Inclusion criteria:

- Confirmed diagnosis of IC according to established diagnostic criteria.^6^
- Age 30~74 years.
- Completion of cystoscopic and urodynamic evaluation before treatment, with cystoscopic findings of Hunner lesions or mucosal glomerulations.
- Urodynamic evidence during the filling phase of a maximum bladder capacity <200 mL accompanied by spontaneous or provoked uninhibited detrusor contractions.
- Symptom duration exceeding six weeks.
- A completed three days voiding diary demonstrating a normal 24 hours urine volume.
- Lack of response to prior antibiotic therapy.
- Absence of psychiatric or neurological disorders that might interfere with study participation.
- Provision of informed consent and willingness to comply with study procedures.
- Complete clinical and follow-up data.
- No known hypersensitivity or contraindication to the medications used in this study.

### Exclusion criteria:

- Coexisting urinary tract conditions, including infection, malignancy, tuberculosis, or obstructive uropathy.
- Other urological disorders such as acute urinary retention, urinary incontinence, neurogenic bladder, chemical cystitis, or radiation-induced cystitiss.
- A prior history of chronic systemic conditions including fibromyalgia, Sjögren’s syndrome, migraine, irritable bowel syndrome, endometriosis, or hypertension.
- Previous surgery involving the bladder, pelvis, or urethra.
- Inability to complete the prescribed treatment protocol.
- Significant dysfunction of major organs or coagulation disorders.
- Documented allergy to any medication used in the present study.

### Treatment Methods:

After completion of preoperative evaluations and exclusion of surgical contraindications, all patients underwent cystoscopic examination followed by bladder hydrodistension. The procedures were detailed as follows: Under general anesthesia, patients were placed in the lithotomy position. With the irrigation fluid reservoir positioned 80 cm above the level of the pubic symphysis (corresponding to approximately 80 cmH_2_O intravesical pressure), a cystoscope was introduced transurethrally.

The bladder walls were systematically inspected for masses, diverticula, calculi, ulcerations, and hemorrhagic lesions, and random biopsies were obtained when indicated. Bladder filling was continued until further inflow of irrigation fluid became minimal, at which point the intravesical pressure was estimated to have reached approximately 80 cmH_2_O. In female patients, given that the urethral resistance is lower, leakage of irrigation fluid around the cystoscope may occur before the target pressure is achieved.

In such cases, digital vaginal compression was applied to reduce peri-instrument leakage and ensure attainment of the predefined intravesical pressure, thereby improving the accuracy of bladder capacity assessment. In a subset of patients with severe IC, marked mucosal bleeding or tearing developed before the target pressure was reached, necessitating premature termination of hydrodistension. Once 80 cmH_2_O was achieved, the bladder was maintained at this pressure for 1–3 min, after which the fluid was drained. The bladder was subsequently refilled to reassess the mucosa. The presence of more than 10 petechial hemorrhages per quadrant involving at least three quadrants was considered indicative of positive glomerulation. For patients without ulcerative lesions or prominent erythema, hydrodistension alone was performed. In contrast, patients with Hunner-type lesions or obvious inflammatory erythema underwent concomitant transurethral resection or electrocauterization of the affected areas during the same procedure. All the surgeries were performed and followed by the same group of surgeons assigned for this study.

Patients in the control group received oral mirabegron sustained-release tablets at a dose of 50 mg once daily for six consecutive months in addition to hydrodistension. In the study group, intravesical instillation of sodium hyaluronate (Cystistat, 40 mg/50 mL) was administered in addition to the control regimen. A total of 40 mg sodium hyaluronate was instilled into the bladder and retained for at least 30 min, with patients encouraged to prolong retention whenever feasible. The instillation schedule consisted of once weekly for the first four weeks, once every two weeks for the subsequent eight weeks, and thereafter once monthly for four months, yielding a total treatment duration of six months.

### Outcome measures:

1.3.1 Clinical symptom improvement: Clinical symptoms were evaluated before and after treatment using the Pelvic Pain and Urgency/Frequency (PUF) Patient Symptom Scale, Interstitial Cystitis Symptom Index (ICSI), and Interstitial Cystitis Problem Index (ICPI). The PUF scale primarily assesses pelvic pain and urinary frequency, with a maximum score of 35, and higher scores indicate greater symptom severity. The ICSI evaluates the severity of symptoms such as nocturia and bladder/pelvic pain, with a maximum score of 20 and higher scores corresponding to more severe symptoms. The ICPI reflects the degree to which IC/BPS-related symptoms affect patients’ daily life, with a maximum score of 16 and higher scores representing greater perceived distress. 1.3.2 Clinical efficacy: Therapeutic response was categorized as follows: *Significant response (SR):* Reduction in ICSI and ICPI scores >20%, decrease in voiding frequency >50%, and significant alleviation or complete resolution of pain;

### Partial response (PR):

Reduction in ICSI and ICPI scores >10%, decrease in voiding frequency <50%, and partial pain relief;

### No response (NR):

Reduction in ICSI and ICPI scores <10%, with no notable improvement in urinary frequency or pain. The overall response rate (ORR) was calculated as follows: ORR = (SR cases + PR cases) / total cases × 100%.^7^ 1.3.3 Voiding parameters and bladder capacity: Daytime voiding frequency, nocturia episodes, and maximum bladder capacity were recorded before and after treatment in both groups. 1.3.4 Urinary biomarkers: Urine samples were collected before and after treatment. Levels of epidermal growth factor (EGF), nerve growth factor (NGF), and insulin-like growth factor-1 (IGF-1) were determined using enzyme-linked immunosorbent assay kits according to the manufacturers’ instructions. 1.3.5 Adverse events: Treatment-related adverse events were observed and compared between the two groups.

### Statistical analysis:

All statistical analyses were performed using SPSS 20.0. Continuous variables were presented as mean ± standard deviation (*x̅*±*s*). Between-group comparisons were conducted using the independent-samples *t*-test, whereas within-group comparisons were analyzed using the paired *t*-test. Percentages were compared using the chi-square (χ²) test. A *p*-value of <0.05 was considered statistically significant.

## RESULTS

The study group comprised nine males and 36 females, aged 33–74 years (mean 53.52 ± 20.46 years). The control group included 11 males and 34 females, aged 30–74 years (mean 53.38 ± 20.35 years). Baseline characteristics did not differ greatly between the two groups (all *P >* 0.05), indicating adequate group comparability (Table-I).

**Table-I:** Comparison of baseline characteristics between the study and control groups (*x̅*±*s*, n = 45 per group).

Parameter	Study Group	Control Group	t/χ²	p-value
Age (years)	53.52±20.46	53.38±20.35	0.03	0.97
Male (n)	9	11	0.26	0.61
Disease duration (months)	34.50±5.62	32.85±6.58	1.28	0.21

p > 0.05.

At baseline, there were no statistically significant differences between the two groups in PUF, ICSI, or ICPI scores (all *p >* 0.05). Following six months of treatment, all three scores decreased in both groups. Notably, the reductions were significantly more pronounced in the study group than in the control group (all *p =* 0.00) (Table-II).

**Table-II:** Comparison of pre- and post-treatment symptom scores between the two groups (*x̅*±*s*, n = 45 per group).

Parameter		Study Group	Control Group	t	p-value
PUF	Pre-treatment	24.85±2.37	24.53±2.28	0.65	0.51
	Post-treatment*	11.16±1.12	14.26±2.05	8.91	0.00
ICSI	Pre-treatment	16.93±2.18	16.70±2.24	0.49	0.62
	Post-treatment*	9.35±0.97	11.68±1.57	8.46	0.00
ICPI	Pre-treatment	13.42±1.42	13.50±1.36	0.27	0.78
	Post-treatment*	8.67±0.56	9.52±0.84	5.65	0.00

* p < 0.05.

The evaluation of clinical efficacy demonstrated a significantly higher ORR in the study group compared with the control group (98% *vs*. 84%). The between-group difference reached statistical significance (*P =* 0.03) (Table-III).

**Table-III:** Comparison of clinical efficacy between the two groups (*x̅*±*s*, n = 45 per group).

Group	SR	PR	NR	ORR
Study group	32	12	1	44(98%)
Control group	29	9	7	38(84%)
*χ^2^*				0.94
*p-value*				0.03

p < 0.05.

Before treatment, no significant differences were observed between the two groups in daytime voiding frequency, nocturia episodes, or maximum bladder capacity (all *p >* 0.05). After treatment, patients in the study group exhibited significantly fewer daytime voids and nocturnal episodes compared with the control group (both *p =* 0.00). In addition, maximum bladder capacity increased in both groups, with significantly greater improvement observed in the study group (*p =* 0.00) (Table-IV).

**Table-IV:** Comparison of pre- and post-treatment voiding parameters and bladder capacity between the two groups (*x̅*±*s*, n = 45 per group).

Parameter		Study Group	Control Group	t	p-value
Daytime voiding frequency	Pre-treatment	35.20±3.86	35.50±3.97	0.36	0.72
	Post-treatment*	18.42±2.27	20.31±2.51	3.75	0.00
Nocturia episodes	Pre-treatment	14.33±1.52	14.25±1.60	0.24	0.81
	Post-treatment*	6.70±1.12	8.74±1.20	8.34	0.00
Maximum bladder capacity (mL)	Pre-treatment	125.37±12.50	123.26±12.36	0.81	0.42
	Post-treatment*	215.47±17.22	202.38±18.57	3.47	0.00

* p < 0.05.

Baseline urinary levels of EGF, NGF, and IGF-1 did not differ significantly between groups (all *p >* 0.05). After treatment, the levels of all three biomarkers decreased in both groups, with significantly greater reductions observed in the study group (all *p =* 0.00) (Table-V).

**Table-V:** Comparison of urinary biomarker levels before and after treatment (*x̅*±*s*, n = 45 per group).

Biomarker		Study Group	Control Group	t	p-value
EGF (ng/mL)	Pre-treatment	16.73±2.27	16.82±2.55	0.18	0.86
	Post-treatment*	9.05±0.83	10.69±1.22	7.46	0.00
NGF (ng/L)	Pre-treatment	187.47±17.63	189.05±21.08	0.39	0.70
	Post-treatment*	120.78±14.63	130.59±15.04	3.14	0.00
IGF-1 (pg/mL)	Pre-treatment	25.18±2.10	24.85±2.04	0.76	0.45
	Post-treatment*	12.46±2.31	15.87±2.42	6.65	0.00

* p < 0.05.

The overall incidence of adverse events was 9% (4/45) in the study group and 4% (2/45) in the control group, indicating no statistically significant difference between the two groups (*p =* 0.40) (Table-VI).

**Table-VI:** Comparison of adverse events between the two groups (*x̅*±*s*, n = 45 per group).

Group	Bladder pain	Urinary tract infection	Allergy	Rash	Hypertension	Dry mouth	Incidence
Study group	2	1	0	0	0	1	4(9%)
Control group	1	0	0	0	0	1	2(4%)
χ^2^							0.71
*p*-value							0.40

p > 0.05.

## DISCUSSION

In the present study, patients without ulcerative lesions or significant erythema underwent hydrodistension alone, whereas those with Hunner lesions or marked inflammatory changes received concomitant transurethral resection and electrocauterization. Symptom relief was observed across all subgroups, including those with ulcerative or erythematous lesions. Notably, the most pronounced improvement occurred in the early post-procedural period, whereas long-term durability was limited, consistent with previous studies. Mirabegron is a novel, highly selective β3-adrenergic receptor agonist that acts on β3 receptors in the detrusor muscle, inducing relaxation during the storage phase and thereby reducing detrusor overactivity and contraction frequency.^8^ Compared with muscarinic receptor antagonists, mirabegron demonstrates better long-term tolerability and adherence. It has been shown to effectively reduce urinary frequency, urgency, and nocturia while increasing functional bladder capacity. Dey A et al.^9^ further suggested that, compared with solifenacin and other agents, mirabegron more closely preserves physiological voiding dynamics and does not impair bladder emptying. However, prolonged monotherapy with mirabegron is unlikely to adequately relieve chronic pelvic pain, possibly because pharmacological modulation of detrusor activity alone cannot reverse structural defects of the urothelial barrier or fully suppress ongoing mucosal irritation.

The GAG layer covering transitional epithelial cells functions as a critical barrier against urinary microorganisms, carcinogens, microcrystals, and other irritants. It is widely regarded as a principal defense mechanism that protects the urothelium from urinary stimulants.^10^,^11^ Clinically, patients typically present with suprapubic or perineal pain that partially subsides after voiding, accompanied by daytime urinary frequency (≥8 voids per day) and nocturia (≥2 episodes per night). A subset of patients exhibit Hunner lesions or diffuse submucosal glomerulations. Because no specific diagnostic biomarker has yet been established, IC remains a diagnosis of exclusion, and many patients experience a protracted clinical course before definitive diagnosis.^12^ Cystoscopic findings following hydrodistension under anesthesia often reveal characteristic petechial hemorrhages or Hunner lesions. Based on cystoscopic appearance, IC is commonly classified into ulcerative (Hunner-type) and non-ulcerative subtypes. The ulcerative form is characterized by one or more discrete ulcers located on the bladder base or lateral wall, with a reported detection rate of approximately 10%. In contrast, the non-ulcerative form, observed in nearly 90% of patients, typically presents with diffuse mucosal glomerulations after hydrodistension.^13^ Although these cystoscopic features are considered supportive of the diagnosis. A definitive diagnosis of IC requires exclusion of other conditions that may produce similar clinical or endoscopic findings, including urinary tract infection, bladder malignancy, eosinophilic cystitis, malakoplakia, schistosomiasis, and detrusor endometriosis.

Currently, IC management generally follows a stepwise paradigm, encompassing both conservative and surgical approaches. First-line therapy primarily consists of behavioral intervention (e.g., bladder training, dietary adjustment) and oral medication (e.g., antihistamines, antidepressants). However, the overall symptom relief rate remains below 30%. Second-line interventions include bladder hydrodistension, which enhances bladder compliance through mechanical stretching. In the short term, approximately 50% of patients experience a ≥50% reduction in pain scores following hydrodistension. Nevertheless, sustained benefit rarely extends beyond six months, and repeated procedures may exacerbate mucosal injury. Third-line options, such as sacral neuromodulation, are associated with substantial cost and carry risks including infection and electrode displacement. More importantly, most currently available treatments target a single pathogenic pathway. For example, anticholinergic agents primarily inhibit detrusor contractions, without addressing the complex interplay among the nerve system, immune system, and epithelial barrier. Consequently, durable therapeutic efficacy remains difficult to achieve. At present, there is no curative therapy for IC. Treatment goals focus on symptom relief and improvement of quality of life. Given the high recurrence rate, combination therapy is often advocated in clinical practice.

Several studies have demonstrated that bladder hydrodistension can alleviate clinical symptoms.^14^ The procedure is considered to exert its therapeutic effect by mechanically disrupting aberrant nerve terminals and promoting neovascularization. Walker SJ et al.^15^ reported that repeated hydrodistension remained effective in patients with symptom recurrence, and that resection of concomitant Hunner lesions provided additional symptomatic improvement. Other investigations^16^ have shown that hydrodistension combined with electrocauterization reduces nocturia episodes. In patients with ulcerative disease, the combination of hydrodistension and lesion resection appears superior to lesion resection alone.

Our findings demonstrate that the proposed triple-modality approach (i.e., bladder hydrodistension combined with oral mirabegron and intravesical instillation of sodium hyaluronate) resulted in significantly greater reductions in PUF, ICSI, and ICPI scores compared with the control regimen (all *p =* 0.00). Hyaluronic acid is a distinctive linear polysaccharide composed of repeating disaccharide units of N-acetyl-D-glucosamine and D-glucuronic acid arranged in alternating linkage.^17^ Sodium hyaluronate is believed to restore defects in the urothelial GAG layer, thereby re-establishing the mucosal barrier. By limiting the transurothelial diffusion of toxic urinary solutes into the detrusor layer, it mitigates chemical irritation and reduces stimulation of subepithelial nerve endings, ultimately improving clinical symptoms Previous studies have reported favorable outcomes with intravesical instillation of sodium hyaluronate in patients with IC.^18^

In the present cohort, the ORR was significantly higher in the study group than in the control group (*p =* 0.03). Moreover, daytime voiding frequency and nocturia episodes were further reduced, and maximum bladder capacity was more substantially increased in patients receiving the combination therapy (all *p =* 0.00). Importantly, the incidence of adverse events did not differ significantly between groups (*p =* 0.40). The favorable tolerability profile may be attributed to the intrinsic biological properties of hyaluronic acid, including its non-immunogenic, non-sensitizing, and non-pyrogenic characteristics, which translate into minimal adverse effects when administered intravesically. Patients with IC typically have reduced functional bladder capacity, and pharmacotherapy alone often yields suboptimal results. Hydrodistension transiently increases bladder capacity, while the addition of mirabegron enhances detrusor stability during the storage phase, prolonging bladder filling time. This extended storage interval may facilitate adequate retention of intravesically instilled sodium hyaluronate, thereby prolonging therapeutic benefit, alleviating related symptoms, and improving bladder capacity.

Experimental data from animal models have shown that the administration of hyaluronic acid not only prolongs inter-voiding intervals and increases maximum bladder capacity, but also significantly reduces urinary expression of inflammatory mediators, including cluster of differentiation three, intercellular adhesion molecule-1, tumor necrosis factor-α, interferon-γ, interleukin-1β, interleukin-6, and transient receptor potential cation channel subfamily M (melastatin) member 8(all *p<* 0.01).^19^ Consistent with these findings, our study demonstrated that patients receiving intravesical sodium hyaluronate exhibited significantly more pronounced reductions in urinary EGF, NGF, and IGF-1 levels compared with the control group (*p =* 0.00). A previous study^20^ has indicated that multiple growth factors (*e.g*., EGF, NGF, insulin-like growth factor-4, and their regulatory proteins) are actively involved in urothelial proliferation and differentiation and are closely associated with the maintenance of normal bladder epithelial function. The triple-modality regimen in the current study may improve the intravesical microenvironment, facilitate tissue repair at sites of mucosal damage, normalize urothelial function, and mitigate inflammatory responses, thereby reducing the production of EGF, NGF, and IGF-1.

### Limitations:

The inclusion criteria were relatively stringent, and the overall treatment cost was comparatively high, resulting in a limited sample size of eligible and adherent participants. In addition, the follow-up period of six months may be insufficient to fully assess long-term durability. To generate higher-level evidence capable of guiding long-term clinical decision-making, future studies should expand the sample size, extend the follow-up duration, and adopt prospective randomized controlled designs.

## CONCLUSIONS

The proposed triple-modality approach can enhance clinical efficacy, alleviate IC-related symptoms, reduce voiding frequency, increase bladder capacity, and lower urinary levels of EGF, NGF, and IGF-1. Notably, such therapeutic benefit can be achieved without a significant rise in adverse events, making this regimen a safe and clinically beneficial option for the affected population. Therefore, the combination therapy offers valuable insights into the individualized treatment of IC.

### Authors’ Contributions:

**LD** and **ZM:** Conceived, designed the study and final approval of the manuscript, are responsible and accountable for the accuracy or integrity of the work. **WX** and **WL:** Collected the data, performed the analysis, critical review, were involved in the writing of the manuscript. All authors read and approved the final manuscript.
